# Indirect Treatment Comparison of ARIA Outcomes for Lecanemab Compared to Donanemab Based on Reported Results

**DOI:** 10.1002/alz70860_103048

**Published:** 2025-12-23

**Authors:** Marwan N. Sabbagh, Sharon Cohen, Ashley Holub, Keith A. Betts, Shumin Rui, Christopher H van Dyck, Anna Burke

**Affiliations:** ^1^ Barrow Neurological Institute, Phoenix, AZ, USA; ^2^ Toronto Memory Program, Toronto, ON, Canada; ^3^ Analysis Group, Boston, MA, USA; ^4^ Analysis Group, Los Angeles, CA, USA; ^5^ Yale School of Medicine, New Haven, CT, USA

## Abstract

**Background:**

Although both anti‐amyloid monoclonal antibodies have demonstrated slowed disease progression in mild cognitive impairment and mild dementia due to Alzheimer's disease, lecanemab and donanemab have different mechanisms of action and, consequently, safety profiles. This study conducted indirect treatment comparisons (ITCs) of ARIA outcomes and death to understand safety differences.

**Method:**

This ITC utilized ARIA rates in the FDA‐approved labels, FDA review documents, or peer‐reviewed publications. Outcomes included ARIA with edema/effusions (ARIA‐E), ARIA with microhemorrhage or superficial siderosis (ARIA‐H), intracerebral hemorrhage (ICH, ≥1cm), APOE4 status, all‐cause death, and other ARIA outcomes in the overall clinical trial population over 18 months. Anchored Bucher ITCs were used to compare rates of ARIA outcomes between lecanemab and donanemab, leveraging placebo as the common comparator. Risk difference‐in‐difference (RDD), 95% confidence intervals (CI), and *p*‐values for normal distribution comparing treatments were derived.

**Result:**

Lecanemab demonstrated significantly lower risk of any ARIA compared to donanemab (ARIA‐E or ARIA‐H) with a RDD of ‐10.1% (95% CI: [‐15.3%, ‐5.0%]). Similarly, the RDD for ARIA‐E (‐10.7% [‐14.5%, ‐6.9%]) and ARIA‐H (‐10.1% [‐15.0%, ‐5.2%]), were also significantly lower. Among APOE4 carriers, these differences persisted for ARIA‐E (‐11.1% [‐16.0%, ‐6.2%]), and ARIA‐H (‐14.4% [‐20.5%, ‐8.2%]). Lecanemab also had significantly lower risk of microhemorrhage (‐7.5% [‐12.1%, ‐2.9%]), and superficial siderosis (‐8.8% [‐12.0%, ‐5.6%]). Incidence of ICH (0.3% [‐0.5%, 1.1%]) and death (‐1.0% [‐2.4%, 0.5%]) were not significantly different between treatments. Lecanemab demonstrated significantly lower risk of death with concurrent ARIA or ICH (‐0.5%, [‐0.9%, 0.0%]) compared to donanemab.

**Conclusion:**

Lecanemab demonstrated significantly lower risks of ARIA outcomes overall and in subgroups of interest compared to donanemab, including death related to ARIA or ICH, in ITC analysis. Future research understanding comparative safety profiles in real‐world settings may inform treatment decisions.

